# 

**Published:** 1877-06

**Authors:** 


### ARTICLE II.

### DENTAL ASSOCIATIONS.

*The South Carolina State Dental Association.*

The State Board of Dental Examiners will meet in the city of Columbia, S. C., commencing at 9 o'clock, P. M., Tuesday, June 5th, 1877. A cordial invitation is extended to all in the profession to attend this meeting. "Come, and let us reason together."

*President*—Dr. J. W. Norwood. *Vice-President*—Dr. J. R. Thompson. *Corresponding Secretary*—Dr. H. D. Wilson. *Recording Secretary*—Dr. G. F. S. Wright. *Treasurer*—Dr. T. W. Bourcher.

*Executive Committee*—Drs. G. F. S. Wright, T. T. Moore. D. L. Boozer, D. R. McCallum, G. B. White.

*Membership*—Drs. W. S. Brown, H. D. Wilson, H. B. Rice, J. Q. McDavid, O. J. Bond.

*Operative Dentistry*—Drs. J. B. Patrick, J. R. Thompson, B. A. Muckenfuss, B. H. Teague, T. Berwick Legare.

*Mechanical Dentistry*—Drs. J. S. Thompson, C. C., Patrick, I. H. Alexander, S. M. Dinkins, W. S. Reynolds.

The following subjects are suggested for Essays and Discussions: 1. Dental Education. 2. Causes of Deterioration. 3. The Effects of Dead Teeth on the System. 4. Best Materials for Filling Roots of Teeth. 5. Separating Teeth. 6. Decidious Teeth—their relation to the permanent, and their treatment. 7. Irregularities of Permanent Teeth—their Cause and Treatment. 8. Relative Merits of Cohesive and non-Cohesive Gold as a Filling for Teeth. 9. Treatment of Exposed Nerves, Dead Teeth, and Alveola Abscess. 10. When to Extract. 11. The Best Base. 12. Selection of Artificial Teeth.

---

### *Pennsylvania State Dental Society.*

The ninth annual meeting of this Society will convene at Minequa Springs, Tuesday, July 10th, at 10 o'clock A. M.

The essayists and subjects will be announced in the next number of this journal. It is designed to have surgical as well as operative clinics; dentists having cases suitable for surgical clinics, will confer a favor by corresponding with the Executive Committee at once.

Minequa is 40 miles North of Williamsport, on the Allegheny Range, a delightfully cool and pleasant summer resort.

The hotel rates to persons attending the Society will not exceed $2.00 per day; ample arrangements are being made for the comfort and accommodation of all who will attend. Rooms can be secured by addressing W. D. Tyler, Superintendent, Minequa, Pa., or the undersigned. Members of the dental profession from this and other states are invited to be present—Excursion tickets can be obtained from all principal points.

G. W. Klump, *Chairman Executive Committee,*
Williamsport Pa.

---

### *The American Dental Association.*

The Annual Meeting of this Association will be held in Chicago, August 7th, 1877.

The following are the Standing Committees:

*Physiology.*—M. S. Dean, R. B. Winder, J. N. Farrar.

*Pathology and Surgery.*—I. Knapp, J. Taft, F. H. Rehwinkel.

*Histology and Microscopy.*—C. N. Pierce, E. D. Swain, H. A. Longnecker.

*Chemistry.*—S. B. Palmer, J. S. Cassidy, C. J. Essig.

*Therapeutics.*—C. A. Brackett, C. R. Butler, F. M. Odell.

*Operative Dentistry.*—G. H. Cushing, A. W. Harlan, F. J. S. Gorgas.

*Mechanical Dentistry.*—W. N. Morrison, F. A. Hunter, M. W. Foster.

*Etiology.*—D. C. Hawxhurst, M. H. Webb, H. L. Sage.

*Literature.*—W. A. Bronson, A. L. Northrop, E. A. Bogue.

*Education.*—L. Jack, J. N. Crouse, Thos. Fillebrown.

*Prize Essays.*—W. H. Morgan, B. Lord, L. D. Shepard.

C. Stoddard Smith, Secretary.

---

### *The Tennessee Dental Association.*

The Annual Meeting of this Association will be held at Lookout Mountain, commencing Wednesday, June 27th, at 10 o'clock, A. M.

Reduced rates by rail and reduced hotel charges have been secured, and all are invited to attend.

L. G. Noel, Secretary.

---

### *The Southern Dental Association.*

In accordance with a resolution passed at the last Annual Meeting of "The Southern Dental Association," held at Montgomery, Ala., requesting the Presiding Officer to call a special session, I hereby notify all members of the above mentioned Society, that there will be a Special Session held at Deer Park, Md., commencing Tuesday, Aug. 14th, 1877.

S. J. Cobb, President,
E. S. Chisholm, Rec. Secretary.

### TO READERS AND CORRESPONDENTS.

Communications intended for publication, and exchange journals and papers, should be addressed to the EDITOR OF THE AMERICAN JOURNAL OF DENTAL SCIENCE, 259 N. Eutaw St., Baltimore, Md. All letters relating to the business of the journal, or containing cash remittances, to be directed to Messrs. SNOWDEN & COWMAN. Articles for publication should be received by the editor at least three weeks previous to the first day of the month on which the number in which it is designed for them to appear, is issued.

☞The American Journal of Dental Science will contain fifty pages of reading matter in each number, making a volume of about

*Six Hundred* pages

EXCLUSIVE OF ADVERTISEMENTS

THE

### AMERICAN JOURNAL

OF

### DENTAL SCIENCE.

F. J. S. GORGAS, M. D., D. D. S., Editor.

The *Journal* is issued on the first of each month and contains not less than *fifty* pages of reading matter in each number, exclusive of advertisements, making a volume of *six hundred* pages per annum.

In each number will be found Original Communications, Correspondence, Selected Articles, Monthly Summary, Bibliographical Notices and Editorial Matter, and every effort will be exerted to render the *Journal* worthy of the patronage of the Dental profession.

A generous co-operation is respectfully solicited from the members of the profession; and as the *Journal* has now a printing office of its own, which materially lessens the cost of its publication, it will be issued at the reduced subscription price of

**$2.50 Per Annum in Advance.**

Communications are respectfully solicited on all subjects pertaining to the practice of dentistry, as well as reports of cases occurring in dental practice.

### RATES OF ADVERTISING.

| | 1 MO. | 2 MO. | 3 MO. | 6 MO. | 12 MO. |
|---|---|---|---|---|---|
| 1 PAGE. | $15 00 | $22 50 | $30 00 | $50 00 | $100 00 |
| ½ " | 10 00 | 15 00 | 20 00 | 30 00 | 50 00 |
| ¼ " | 7 00 | 11 00 | 15 00 | 20 00 | 30 00 |
| ⅛ " | 5 00 | 8 00 | 11 00 | 15 00 | 20 00 |

All original communications designed for publication, works for review, new instruments for notice, exchanges, &c., should be directed to the editor, 259 N. Eutaw St., Baltimore, Md.

All advertisements and subscriptions should be addressed to

SNOWDEN & COWMAN,

*Publishers of the Am. Journal of Dental Science.*

86 W. FAYETTE ST. BET. CHARLES & LIBERTY STS.,

BALTIMORE, MD.

